# Organoid technology in cancer research

**DOI:** 10.1186/s43556-026-00505-5

**Published:** 2026-06-30

**Authors:** Jingjing Zhang, Jian He

**Affiliations:** Center for Single-Cell Omics, School of Public Health, Shanghai Jiao Tong University School of Medicine, Shanghai, China

**Keywords:** Tumor organoids, Patient-derived organoids, Precision oncology, Immuno-oncology, Drug discover

## Abstract

Organoid technology has emerged as a transformative tool in cancer research by recapitulating the structural complexity, genomic integrity, and phenotypic heterogeneity of human primary tumors. Over the past 15 years, this technology has transcended the limitations of traditional preclinical cancer models, evolving from a specialized developmental biology technique into a versatile platform that spans basic mechanistic research, translational drug development, and clinical personalized medicine. This review provides a comprehensive and in-depth overview of organoid systems, spanning fundamental biological principles, standardized methodologies for tumor organoid establishment, and multi-dimensional applications across the entire cancer research continuum. We highlight mechanistic insights into tumor initiation, clonal evolution, progression, and metastatic colonization revealed by organoid models, as well as translational advances in high-throughput drug discovery, functional precision oncology, immuno-oncology therapeutic development, and early cancer detection and interception. Particular emphasis is placed on therapeutic development strategies and recent prospective clinical trials validating patient-derived organoids (PDOs) as predictive functional biomarkers for treatment response, which represents the most critical translational milestone in the field. By integrating cutting-edge single-cell multi-omics technologies, spatial profiling, and bioengineering advances, we further discuss current limitations, unresolved technical and biological challenges, and future directions aimed at bridging the long-standing gap between bench research and clinical practice. This work aims to present an updated, clinically oriented synthesis of organoid applications in oncology, with the ultimate goal of accelerating the clinical translation of organoid technology to advance precision cancer care and improve patient outcomes.

## Introduction

Cancer remains a global health challenge characterized by high mortality rates and adaptive resistance to current targeted and immune therapies [1, 2]. One of the main bottlenecks in overcoming these clinical obstacles is the lack of preclinical models that can truly replicate the biological characteristics of human tumors in vivo [3]. Traditional two-dimensional (2D) cell lines often fail to predict clinical efficacy due to the loss of tissue structure, clonal diversity, and extensive genetic drift during long-term culture [4]. Patient-derived xenograft (PDX) models can better preserve the tumor structure, but their application is limited by several factors. These limitations include high cost, low throughput, the occurrence of murine stromal replacement, and the need to use immunodeficient hosts, which prevent researchers from conducting studies on the interaction between autologous tumors and the immune system [5, 6].

In 2009, the Clevers research group successfully constructed the first intestinal organoids derived from adult stem cells [7]. Since then, organoid technology has expanded into the fields of cancer modeling and translational research [8]. Organoids are three-dimensional (3D) structures formed by the self-assembly of adult stem cells (ASCs) or pluripotent stem cells (PSCs), and they can to some extent reproduce the structure and function of natural tissues, cell-to-cell interactions, and biological complexity [9]. In the field of oncology, tumor-derived organoids, often referred to as patient-derived organoids (PDOs), are cultivated from biopsy or surgical specimens [10]. Tumor organoids overcome the key limitations of two-dimensional culture and PDX models. During multiple passages, tumor organoids can not only maintain the genomic characteristics, histopathological status, and clonal heterogeneity of the primary patient tumor, but also support long-term stable expansion, cryopreservation, and high-throughput experimental operations [11–13]. These characteristics make organoids a transformative platform for understanding cancer biology and developing personalized treatment strategies.

Although there are numerous works in this field, the existing reviews are often limited to specific cultivation protocols or a single type of cancer, thus leaving a gap in comprehensively evaluating tumor organoids from mechanism analysis to clinical validation [14, 15]. At the same time, organoid technology is rapidly integrating with immune-stromal co-cultures, liquid biopsies, and advanced computational tools, which requires us to provide a more novel and clinical-oriented comprehensive review [16, 17]. Currently, what needs to be evaluated is how these high-fidelity models bridge the long-standing gap between basic research and precision oncology, especially when they are combined with single-cell multi-omics and artificial intelligence (AI) technologies [18]. In response to this demand, this review assesses the milestones of organoid technology in translational applications and focuses on its predictive capabilities in the clinical environment.

This review systematically summarizes the development and clinical application of organoids technology in the field of oncology. It first introduces the basic knowledge of organoids, highlighting their core biological principles, related signaling pathways, and the role of extracellular matrix in the self-assembly process. It then discusses the construction methods of tumor organoids, presenting the standard workflows for establishing organoid models starting from clinical samples and through genetic engineering. It focuses on assessing the main applications of organoid models in cancer research, including disease modeling, drug screening, immuno-oncology, and early diagnosis. It will also objectively analyze the current technical limitations and future development directions in this field. Overall, this review highlights the value of organoid technology in facilitating the transition from basic research to clinical practice, providing a reliable platform for advancing the development of precision oncology.

## Fundamentals of organoid technology

**Fig.1:**
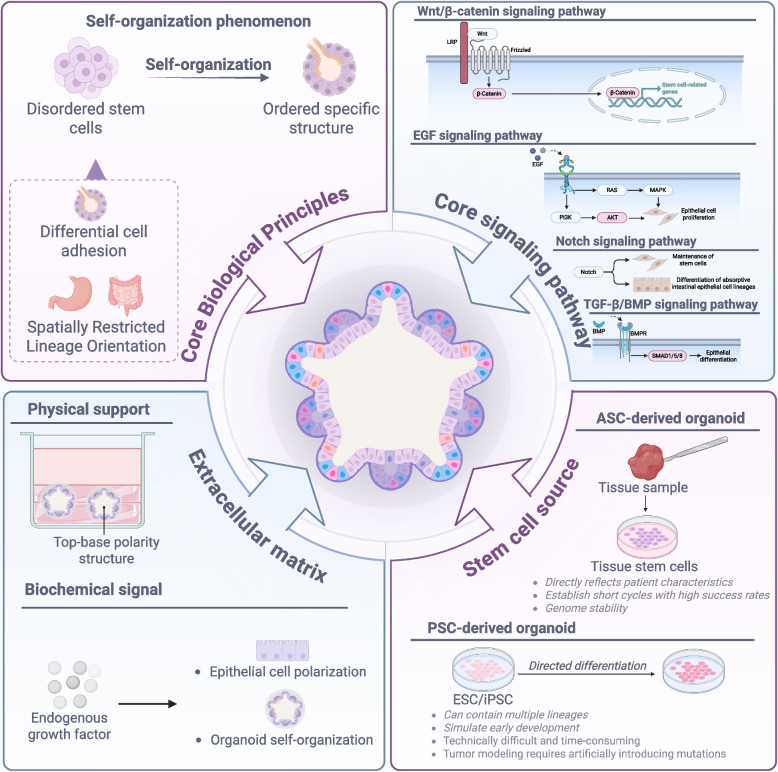
The schematic overview of organoid fundamentals. The generation and maintenance of organoids rely on the complex interplay of four foundational pillars. (1) Stem cell sources: Organoids can be derived from ASCs via tissue biopsy or from PSCs (ESCs/iPSCs) through directed differentiation, each offering distinct advantages for tissue modeling. (2) Core biological principles: The fundamental driver is self-organization, governed by differential cell adhesion and spatially restricted lineage orientation, enabling disordered stem cells to spontaneously form ordered, tissue-specific structures. (3) Core signaling pathways: Evolutionarily conserved niche signals—specifically the Wnt/β-catenin, EGF, Notch, and TGF-β*/*BMP pathways—tightly regulate stem cell self-renewal, proliferation, and multi-lineage differentiation. (4) Extracellular matrix: The ECM provides essential physical scaffolding to establish apical-basal polarity, alongside biochemical cues (endogenous growth factors) necessary for epithelial polarization and structural integrity. Together, these elements enable the in vitro reconstruction of miniature functional organs. ASC: Adult stem cell; PSC: Pluripotent stem cell; ESC: Embryonic stem cell; iPSC: Induced pluripotent stem cell; EGF: Epidermal growth factor; TGF-β: Transforming growth factor-β; BMP: Bone morphogenetic protein; ECM: Extracellular matrix

Organoids transcend simple 3D spheroids by functioning as miniature, self-organized tissue structures that faithfully recapitulate the architecture, cellular diversity, and functional complexity of native human organs [19–21]. Their emergence is a transformative breakthrough rooted in a profound understanding of embryonic organogenesis and adult tissue homeostasis. Rather than random aggregation, organoids undergo programmed self-assembly driven by the intricate interplay of four foundational pillars: the strategic selection of stem cell sources, the inherent core biological principles governing self-organization, the precise modulation of core signaling pathways, and the vital physical and biochemical support provided by the extracellular matrix (ECM) (Fig. 1).

### Core biological principles of organoid self-organization

The defining feature of organoid technology is its reliance on the intrinsic self-organizing capacity of stem cells, a fundamental biological property that drives embryonic development and adult tissue regeneration [22, 23]. Self-organization in organoid cultures refers to the spontaneous formation of ordered, tissue-specific structures from a population of initially disorganized stem or progenitor cells, without the need for external patterning scaffolds or pre-defined structural guidance [24, 25]. This process is driven by two core biological mechanisms: differential cell adhesion and spatially restricted lineage commitment, both of which are tightly regulated by evolutionarily conserved developmental signaling pathways.

Differential cell adhesion, first described by Steinberg in the 1960s, drives the spatial sorting of different cell types within the organoid, leading to the formation of polarized tissue structures such as the inward-facing crypt-like domains in intestinal organoids, or the apical-basal polarity of airway and pancreatic ductal organoids [26, 27]. Spatially restricted lineage commitment, meanwhile, is regulated by paracrine signaling between adjacent cell types within the organoid, creating local signaling gradients that mimic the in vivo tissue niche and direct the differentiation of stem cells into the full complement of functional cell types present in the native organ [28, 29]. For example, in intestinal organoids, Paneth cells located at the base of crypt-like domains secrete Wnt and Notch ligands to maintain the adjacent stem cell population, while the upward migration of cells away from this niche leads to differentiation into absorptive enterocytes and secretory cell lineages, mirroring the cellular hierarchy of the native intestinal epithelium [30].

This intrinsic self-organizing capacity is what makes organoids uniquely capable of mimicking in vivo tissue physiology and pathology, far beyond what is possible with conventional 2D or simple 3D culture systems. In the context of cancer, this self-organizing property is preserved in tumor organoids, which recapitulate the histological architecture, cellular heterogeneity, and polarity of the original patient tumor, including the formation of glandular structures in colorectal and gastric adenocarcinoma organoids, and the squamous differentiation patterns seen in head and neck and esophageal cancer organoids [31, 32].

### Core signaling pathways regulating organoid culture

At its core, organoid technology relies on the precise in vitro replication of the developmental and homeostatic tissue microenvironment (niche) that sustains stem cell stemness and directs physiologically relevant lineage differentiation. ASCs and PSCs, such as embryonic stem cells (ESCs) and induced pluripotent stem cells (iPSCs), embedded in ECM gels respond to a cocktail of growth factors and small molecules that modulate evolutionarily conserved developmental pathways—primarily Wnt/β-catenin, epidermal growth factor (EGF), Notch, and transforming growth factor-β (TGF-β)/bone morphogenetic protein (BMP) signaling [33, 34]. The precise modulation of these pathways is the foundation of successful organoid culture, with different tissue types requiring distinct pathway activation or inhibition to support long-term stem cell expansion while preserving physiological lineage commitment [35].

The Wnt/β-catenin pathway is the master regulator of stem cell self-renewal in most epithelial tissues, and is therefore the most commonly modulated pathway in organoid culture systems [36]. Physiologically, Wnt ligands bind to Frizzled and LRP5/6 receptors, inhibiting the β-catenin destruction complex and leading to nuclear translocation of β-catenin, which activates the transcription of stem cell-associated genes. In most epithelial organoid cultures, Wnt signaling is enhanced through the addition of exogenous Wnt3a and R-spondin 1, a secreted protein that potentiates Wnt signaling by inhibiting the Wnt antagonist RNF43/ZNRF3 [37, 38]. For example, Wnt activation is essential for the maintenance of intestinal, gastric, hepatic, and pancreatic stem cells in organoid culture, with withdrawal of Wnt/R-spondin leading to spontaneous differentiation of stem cells into mature tissue lineages [39, 40].

The EGF signaling pathway is a key regulator of epithelial cell proliferation in organoid cultures, acting via the mitogen-activated protein kinase (MAPK) and phosphoinositide 3-kinase (PI3K)/AKT pathways [41]. EGF is a universal component of nearly all epithelial organoid culture media, supporting the proliferation of both stem and progenitor cell populations. Notably, many epithelial cancers harbor activating mutations in the epidermal growth factor receptor (EGFR)/rat sarcoma (RAS)/MAPK pathway, such as Kirsten rat sarcoma viral oncogene homolog (KRAS) mutations in colorectal and pancreatic cancer, which render tumor organoids independent of exogenous EGF supplementation—a feature that is widely used to selectively enrich for tumor cells in organoid cultures, while suppressing the growth of normal epithelial cells [42, 43].

The Notch pathway is a critical regulator of cell fate determination and lineage specification in epithelial tissues, and plays a central role in maintaining the stem cell pool and directing secretory vs. absorptive lineage differentiation in organoid cultures [44]. In intestinal organoids, for example, activation of Notch signaling promotes the maintenance of stem cells and drives differentiation towards the absorptive enterocyte lineage, while inhibition of Notch signaling leads to massive secretory cell differentiation, particularly into goblet cells [45]. In airway organoids, Notch modulation directs the balance between ciliated and secretory cell lineages, while in pancreatic organoids, Notch signaling supports the maintenance of ductal progenitor cells [46, 47].

The TGF-β/BMP signaling pathway is a key negative regulator of stem cell self-renewal in most epithelial tissues, and its inhibition is therefore essential for long-term organoid culture. BMP ligands, which signal via the SMAD1/5/8 pathway, promote epithelial differentiation and limit stem cell proliferation in the intestinal epithelium, liver, and other tissues [48, 49]. For this reason, the BMP inhibitor Noggin is a core component of most epithelial organoid culture media, preventing differentiation and supporting long-term stem cell expansion [50]. Similarly, inhibition of TGF-β signaling via small molecule inhibitors such as A83-01 or SB431542 is widely used in organoid cultures, particularly for tumor organoids, to prevent epithelial-mesenchymal transition (EMT) and maintain epithelial cell viability [51].

### The role of the extracellular matrix in organoid formation

Beyond serving merely as a physical scaffold for organoid growth, the ECM functions as a critical regulator of cell polarity, migration, signaling, and lineage differentiation, thereby playing an indispensable role in organoid self-organization and homeostasis. The majority of current organoid culture protocols use Matrigel, a solubilized basement membrane extract derived from Engelbreth-Holm-Swarm (EHS) mouse sarcoma, as the ECM scaffold [52, 53]. Matrigel is rich in laminin, collagen IV, entactin, and heparan sulfate proteoglycans, as well as endogenous growth factors, which provide both physical support and biochemical cues that drive epithelial cell polarization and organoid self-organization [54].

When embedded in Matrigel, epithelial stem cells polarize to form apical-basal polarity, with the apical surface facing an internal lumen and the basal surface in contact with the ECM—mirroring the polarity of native epithelial tissues in vivo [55]. This polarization is essential for the formation of tissue-specific structures such as crypt-villus domains in intestinal organoids, and for the maintenance of physiological cell function, such as vectorial secretion in glandular epithelial organoids [56]. The ECM also modulates key signaling pathways via integrin-mediated cell–matrix adhesion, which crosstalks with Wnt, EGF, and TGF-β signaling to regulate stem cell self-renewal, proliferation, and differentiation.

Despite its widespread use, Matrigel has significant limitations, particularly for clinical translation: it is a poorly defined, xenogeneic product with significant batch-to-batch variability in composition and growth factor content, which leads to inconsistent experimental results and limits its use in clinical-grade organoid culture [57, 58]. To address these limitations, significant research efforts have focused on the development of synthetic, fully defined hydrogel matrices for organoid culture [59, 60]. These synthetic matrices, which include polyethylene glycol (PEG)-based hydrogels, hyaluronic acid-based hydrogels, and collagen-based matrices, can be precisely tuned for mechanical stiffness, adhesive ligand composition, and growth factor presentation, enabling the optimization of culture conditions for specific tissue types. Recent studies have demonstrated that fully defined synthetic hydrogels can support the long-term culture of intestinal, hepatic, and pancreatic organoids, with equivalent performance to Matrigel, while eliminating batch variability and xenogeneic contaminants [61]. These advances are critical for the development of clinical-grade organoid culture systems that meet regulatory requirements for use in patient care [62].

### Adult stem cell vs. pluripotent stem cell-derived organoids

Organoid technology is broadly divided into two major platforms based on the cell source: ASC-derived organoids and PSC-derived organoids, each with distinct advantages, limitations, and applications in cancer research [63, 64].

ASC-derived organoids, the most widely used platform in cancer research, are generated from tissue-resident adult stem cells isolated from primary tissue samples, including surgical resections, core needle biopsies, and even minimally invasive liquid biopsy samples [65]. The primary advantage of ASC-derived organoids is their ability to be generated directly from patient normal and tumor tissue, preserving the exact genomic landscape, clonal heterogeneity, and histological features of the original tissue [66]. ASC-derived organoids can be established rapidly (within 1–2 weeks) from patient samples, with high success rates (over 70%–80%) for most common epithelial cancers, including colorectal, gastric, breast, and pancreatic cancer [67]. They also maintain genomic stability over long-term culture, with minimal clonal drift compared to 2D cell lines, making them ideal for personalized oncology applications, where the organoid acts as a functional avatar for the patient's tumor [68]. The primary limitation of ASC-derived organoids is that they are tissue-specific, and can only generate the epithelial lineages of the tissue from which they were derived, with limited ability to form stromal, vascular, or immune cell components of the tumor microenvironment.

PSC-derived organoids, by contrast, are generated from human ESCs or iPSCs via directed differentiation, mimicking the stepwise embryonic development of the target organ. PSCs have unlimited proliferative capacity and can differentiate into any cell type in the human body, enabling the generation of multi-lineage organoids that contain not only epithelial cells, but also stromal fibroblasts, endothelial cells, and even tissue-resident immune cells, which more fully recapitulate the complexity of the native tissue and tumor microenvironment [69]. For example, PSC-derived brain organoids contain multiple neuronal and glial cell types, and have been widely used to model pediatric brain tumors, which are difficult to model with ASC-derived organoids due to limited sample availability [70, 71]. PSC-derived organoids are also uniquely suited for the study of cancer predisposition syndromes, where iPSCs can be generated from patients with germline cancer predisposition mutations, and differentiated into organoids to study the early stages of tumor initiation [72]. The primary limitations of PSC-derived organoids are that they are technically challenging and time-consuming to generate (often taking months of directed differentiation), and that tumor modeling requires the introduction of oncogenic mutations via gene editing, rather than directly capturing the genomic complexity of primary patient tumors [73].

Together, ASC and PSC-derived organoid platforms provide complementary tools for cancer research, with ASC-derived organoids being the primary platform for personalized oncology and patient-specific tumor modeling, and PSC-derived organoids being particularly valuable for studying early tumor initiation, multi-lineage tumor-microenvironment interactions, and rare pediatric cancers.

The construction of organoids relies on the coordinated integration of multiple factors. These factors include the characteristics of stem cells, the regulation of signaling pathways, the principle of intrinsic self-organization, and the physical support provided by the ECM. These biological foundations ensure that the organoid models can effectively reproduce the complexity of in vivo tissues. Based on these fundamental principles, the next step is to apply them to clinical samples to construct high-fidelity and patient-specific tumor models. This step is important for realizing the translational value of organoids.

## Construction of tumor organoids

**Fig. 2:**
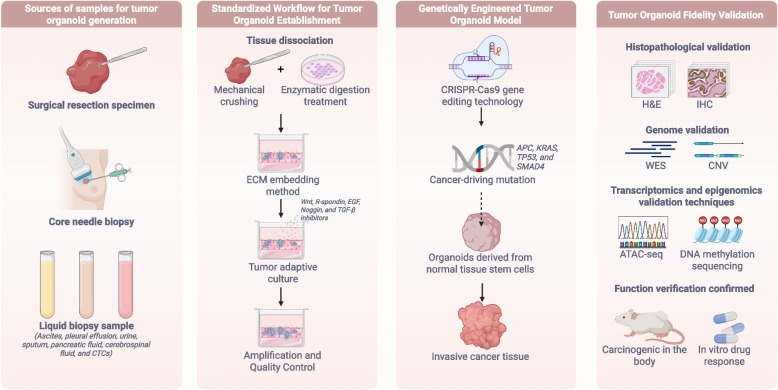
Comprehensive workflow for the establishment, genetic engineering, and validation of tumor organoids. The generation of high-fidelity tumor organoids encompasses four key domains. (1) Sample sources: Starting materials range from traditional surgical resection specimens and core needle biopsies to minimally invasive liquid biopsy samples (e.g., ascites, pleural effusion, and CTCs). (2) Standardized workflow: Tumor tissue undergoes mechanical crushing and enzymatic digestion, followed by ECM embedding. The culture conditions are selectively adapted to favor tumor cell growth over normal epithelium, culminating in stable amplification. (3) Genetic engineering: Alternatively, tumor organoids can be established by introducing canonical cancer-driving mutations (e.g., APC, KRAS, TP53, and SMAD4) into normal tissue stem cell-derived organoids using CRISPR-Cas9 technology to model invasive carcinogenesis. (4) Fidelity validation: To ensure the models accurately recapitulate the primary tumor, rigorous multi-omic and functional validations are required, including histopathological assessment (H&E, IHC), genomic profiling (WES, CNV), transcriptomic/epigenomic sequencing (ATAC-seq, DNA methylation), and functional verification (in vivo tumorigenicity and in vitro drug response). CTC: Circulating tumor cell; ECM: Extracellular matrix; APC: Adenomatous polyposis coli; KRAS: Kirsten rat sarcoma viral oncogene homolog; TP53: Tumor protein p53; H&E: Hematoxylin and eosin; IHC: Immunohistochemistry; WES: Whole-exome sequencing; CNV: Copy number variation; ATAC-seq: Assay for transposase-accessible chromatin using sequencing

The generation of robust, genomically faithful tumor organoids requires the careful adaptation of developmental organoid culture principles to the dysregulated biology of cancer cells. Unlike normal tissue organoids, which require precise supplementation of niche factors to maintain stem cell self-renewal, tumor organoids harbor oncogenic mutations that drive constitutive activation of niche signaling pathways, enabling their growth under selective culture conditions that suppress normal epithelial cell growth. The overarching goal of tumor organoid generation is not maximal growth speed, but rather high fidelity—the preservation of the genomic, transcriptomic, epigenomic, and histopathological features of the parental primary tumor. In this section, we provide a comprehensive overview of the workflow, methodologies, and validation strategies for tumor organoid construction, including sample sources, culture protocols, gene editing approaches, and fidelity validation (Fig. 2).

### Sample sources for tumor organoid generation

Tumor organoids can be successfully generated from a wide range of clinical sample types, spanning from large surgical resection specimens to minimally invasive liquid biopsy samples, each with distinct advantages, success rates, and clinical applications [74]. The choice of sample source depends on the intended application, with surgical samples being preferred for large-scale biobanking, needle biopsies for longitudinal personalized oncology, and liquid biopsy samples for non-invasive disease monitoring and early detection [17].

Surgical resection specimens are the most common source for tumor organoid generation, providing abundant viable tumor tissue with high organoid establishment success rates, often exceeding 80% for colorectal, gastric, breast, and pancreatic cancers [75, 76]. Surgical samples also enable the parallel establishment of paired normal tissue organoids from adjacent non-tumor tissue from the same patient, which are invaluable controls for studying tumor-specific vulnerabilities, drug selectivity, and synthetic lethal interactions [77, 78]. The primary limitation of surgical samples is that they are only available for patients undergoing surgical resection, which excludes the majority of patients with advanced, metastatic, or unresectable disease, who stand to benefit the most from personalized organoid-guided therapy [79].

Core needle biopsies are a minimally invasive sample source that enables the generation of tumor organoids from patients with unresectable primary tumors or metastatic lesions, who are not candidates for surgical resection [80]. Recent studies have demonstrated that core needle biopsies, which typically yield only milligrams of tissue, can be used to generate tumor organoids with high success rates across multiple cancer types, including breast, gastric, prostate, and lung cancer [81]. Critically, needle biopsy-derived organoids preserve the genomic and phenotypic features of the parental tumor, and accurately predict clinical response to chemotherapy and targeted therapy in prospective clinical studies [82]. Needle biopsies also enable longitudinal sampling of a patient's tumor during treatment, allowing the generation of organoids at baseline and at the time of acquired resistance, to study the molecular mechanisms of therapeutic resistance in real time [83].

Liquid biopsy samples, including ascites, pleural effusion, urine, sputum, pancreatic juice, cerebrospinal fluid (CSF), and circulating tumor cells (CTCs) from peripheral blood, represent the most minimally invasive sample source for tumor organoid generation, and have emerged as a transformative platform for non-invasive disease monitoring, early detection, and personalized therapy [84]. Ascites and pleural effusion samples from patients with metastatic abdominal and thoracic cancers have high organoid establishment success rates, and have been widely used to model metastatic disease and predict treatment response [85]. Urine-derived organoids have been successfully generated from patients with bladder and urothelial cancer, enabling non-invasive detection and drug sensitivity testing for this disease [86]. Sputum-derived organoids have been used for the early detection of lung cancer in high-risk smokers, while pancreatic juice-derived organoids enable the early detection of pancreatic cancer, a disease with a dismal 5-year survival rate of less than 10% due to late diagnosis [87, 88]. CTC-derived organoids, while technically more challenging to establish due to the extreme rarity of CTCs in peripheral blood (as few as 1 cell per mL of blood), have been successfully generated from patients with metastatic breast, colorectal, and small cell lung cancer, enabling real-time monitoring of disease evolution and therapeutic response in patients with inaccessible metastatic lesions [89].

### Standardized workflow for tumor organoid establishment

The general workflow for tumor organoid generation consists of four core steps: tissue dissociation, ECM embedding, selective culture in tumor-adapted media, and quality control and validation [76]. While specific culture conditions vary between cancer types, the core workflow is broadly applicable across most solid tumor types.

Tissue dissociation, in which the clinical sample is processed into a single-cell suspension or small tumor fragments (20–50 μm) via mechanical dissociation and enzymatic digestion [90]. For solid tissue samples, mechanical mincing with scalpels or scissors is followed by enzymatic digestion with a combination of collagenases (types I/IV), dispase, and hyaluronidase, which break down the tumor ECM to release viable tumor cells [91]. For liquid biopsy samples, tumor cells are isolated via centrifugation (for ascites/effusion samples) or immunomagnetic enrichment (for CTCs), followed by gentle dissociation into single cells [92]. Critically, the dissociation process must be optimized to maximize cell viability, as excessive enzymatic digestion or mechanical stress can lead to significant cell death and reduced organoid establishment success rates.

ECM embedding, in which the dissociated tumor cells are resuspended in a basement membrane matrix (most commonly Matrigel) and plated as 3D droplets in tissue culture plates, which are allowed to solidify at 37 °C before the addition of culture media. The density of cell embedding is a critical parameter, with optimal seeding densities ranging from 1,000 to 10,000 cells per 50 μL Matrigel droplet, depending on the cancer type and sample viability [93]. For synthetic hydrogel matrices, the embedding process is similar, with cells mixed into the hydrogel precursor solution prior to crosslinking and gelation [94].

Selective culture in tumor-adapted media, which is the most critical step for successful tumor organoid establishment and the selective enrichment of tumor cells over normal epithelial cells. Normal epithelial organoids require a full complement of niche factors, including Wnt, R-spondin, EGF, Noggin, and TGF-β inhibitors, to support stem cell survival and proliferation [95, 96]. Tumor cells, by contrast, frequently harbor oncogenic mutations that constitutively activate these niche signaling pathways, rendering them independent of exogenous growth factor supplementation [97]. This property is exploited to selectively enrich for tumor cells: by removing specific growth factors from the culture media, normal epithelial cells are unable to survive and proliferate, while tumor cells with activating mutations in the corresponding pathway continue to grow, resulting in highly pure tumor organoid cultures. For example, colorectal cancers with biallelic inactivation of the adenomatous polyposis coli (APC) gene, which occurs in more than 80% of colorectal cancers, have constitutive activation of the Wnt pathway, and therefore grow robustly in media without exogenous Wnt or R-spondin, while normal colonic epithelial cells die rapidly in these conditions [98]. Similarly, KRAS-mutant tumor organoids grow independently of exogenous EGF, while tumor protein p53 (TP53)-mutant organoids can survive in the presence of MDM2 inhibitors that kill normal TP53 wild-type cells [42]. This selective conditioning strategy is the gold standard for generating pure tumor organoid cultures, with more than 95% tumor cell purity achieved in most cases, eliminating the need for manual microdissection or fluorescence-activated cell sorting (FACS) of tumor cells.

Tumor organoids typically form within 3–7 days of plating, and can be passaged every 1–2 weeks via mechanical dissociation into small fragments, with stable long-term expansion possible for more than 6 months in most cases. Tumor organoids can also be cryopreserved in liquid nitrogen for long-term banking, with high recovery rates upon thawing, enabling the creation of large living organoid biobanks that span hundreds of patients and dozens of cancer types.

### Gene-engineered tumor organoid models

In addition to patient-derived tumor organoids, gene-engineered tumor organoids represent a powerful complementary platform for cancer research, enabling the precise dissection of the functional consequences of oncogenic mutations, the order of mutation acquisition during tumorigenesis, and synthetic lethal interactions in an isogenic background [99]. These models address a key limitation of patient-derived organoids: the confounding effect of extensive genomic heterogeneity between patient tumors, which makes it difficult to isolate the functional impact of individual mutations.

The most widely used approach for generating gene-engineered tumor organoids is the sequential introduction of cancer driver mutations into normal tissue stem cell-derived organoids using CRISPR-Cas9 gene editing technology, which reconstructs the stepwise evolution of human cancer in a controlled, isogenic system [100]. The landmark 2015 studies from the Clevers group first demonstrated this approach, showing that the sequential introduction of APC, KRAS, TP53, and SMAD4 mutations (the four most common driver mutations in colorectal cancer) into normal human intestinal organoids was sufficient to transform them into invasive carcinoma, which formed metastatic tumors when transplanted into immunocompromised mice [98]. This approach provided the first direct experimental validation of the Fearon-Vogelstein multi-step model of colorectal carcinogenesis, which had been proposed 25 years earlier based on genomic analysis of human tumor samples [101].

Subsequent studies have extended this approach to other cancer types, including gastric, pancreatic, hepatic, lung, and esophageal cancer, defining the order and cooperativity of driver mutations in tumorigenesis across tissues. For example, stepwise editing of TP53, cyclin-dependent kinase inhibitor 2 A (CDKN2A), SMAD4, and KRAS mutations in normal human pancreatic ductal organoids generated invasive pancreatic adenocarcinoma, revealing the cooperative role of these mutations in disrupting pancreatic differentiation and driving malignant transformation [102]. Similarly, editing of EGFR, TP53, and CDKN2A mutations in normal human airway organoids modeled the early stages of lung adenocarcinoma development, identifying critical early events in lung tumorigenesis [103].

Gene-engineered organoids are also uniquely suited for the identification and validation of synthetic lethal interactions, a core therapeutic strategy in precision oncology. Synthetic lethality occurs when the combination of two genetic perturbations results in cell death, while either perturbation alone has no effect, enabling the selective targeting of tumor cells with specific oncogenic mutations [104]. For example, genome-wide CRISPR screening in isogenic organoid models with mismatch repair deficiency identified WRN helicase as a synthetic lethal target in microsatellite-unstable (MSI) cancers, a finding that has since been validated in patient-derived organoids and is now being advanced to clinical trials [105]. Isogenic organoid models have also been used to validate synthetic lethal targets for common oncogenic drivers such as KRAS, APC, and TP53, which have historically been considered undruggable [106].

### Validation of tumor organoid fidelity

Rigorous validation of tumor organoid fidelity to the parental primary tumor is an essential step in organoid generation, as the utility of organoid models for both basic research and clinical applications depends entirely on their ability to faithfully recapitulate the biological features of the original patient tumor. Validation is performed at multiple levels, including histopathological, genomic, transcriptomic, and functional validation, to ensure that the organoid model accurately represents the parental tumor.

Histopathological validation is the first line of fidelity assessment, comparing the hematoxylin and eosin (H&E) staining of organoid sections to the H&E staining of the original parental tumor [107]. Tumor organoids faithfully recapitulate the histopathological features of the original tumor, including glandular formation, nuclear pleomorphism, mitotic index, and differentiation grade, across nearly all solid tumor types [108]. Immunohistochemistry (IHC) is also used to validate the expression of key diagnostic and lineage markers, such as cytokeratins, hormone receptors (ER/PR/HER2 in breast cancer), and mismatch repair proteins, confirming that the organoids maintain the marker expression profile of the original tumor [109].

Genomic validation is the gold standard for confirming that tumor organoids preserve the genetic landscape of the parental tumor. Whole-exome sequencing (WES) and copy number variation (CNV) analysis are routinely used to compare the mutational profile of the organoids to the parental tumor, confirming that the organoids retain the key driver mutations, CNVs, and mutational signatures of the original lesion [110]. Multiple studies have confirmed that early-passage tumor organoids maintain more than 90% genomic concordance with the original primary tumor, preserving both clonal driver mutations and subclonal heterogeneity, including rare subclones linked to therapeutic resistance and early relapse [111]. Long-term culture can lead to minor clonal selection in some cases, but the core driver mutational profile remains stable for more than 20 passages in most tumor organoid lines.

Transcriptomic and epigenomic validation is increasingly used to confirm that organoids preserve the gene expression and epigenetic landscape of the parental tumor. Bulk and single-cell RNA sequencing (RNA-seq) studies have demonstrated that tumor organoids maintain the gene expression profile, molecular subtype, and cellular heterogeneity of the original tumor across multiple cancer types, including breast, colorectal, gastric, and pancreatic cancer [48, 112, 113]. Epigenomic profiling, including assay for transposase-accessible chromatin using sequencing (ATAC-seq) and DNA methylation sequencing, has also shown that tumor organoids preserve the chromatin accessibility and DNA methylation landscape of the parental tumor, including cancer-specific epigenetic alterations that drive oncogenic gene expression [114].

Functional validation confirms that tumor organoids maintain the functional properties of the original tumor, most notably in vivo tumorigenicity and ex vivo drug response. Tumor organoids form tumors in immunocompromised mice that histologically and genomically match the original patient tumor, confirming their tumorigenic potential [97]. Critically, multiple retrospective and prospective clinical studies have confirmed that the ex vivo drug response of patient-derived organoids strongly correlates with the clinical response of the patient to the same therapy, with high positive and negative predictive value, confirming that the organoids maintain the functional therapeutic vulnerabilities of the original tumor [80].

The construction of tumor organoids follows a standardized process, including multiple steps from sample acquisition to multi-dimensional fidelity verification. This process ensures that the organoids are highly consistent with the original tumor at both the genomic and histological levels. This rigorous validation framework not only demonstrates the reliability of organoids as an experimental platform but also lays a solid foundation for their subsequent application in simulating complex disease processes and guiding clinical treatment strategies.

## Applications in cancer research

Over the past decade, organoid technology has expanded into multiple areas of cancer research. These areas include basic research in tumor biology, translational drug development, and clinical personalized oncology. Organoid models can faithfully reproduce the biological characteristics of human tumors in a controllable high-throughput in vitro system. This unique advantage effectively overcomes the long-standing limitations of traditional preclinical models. Therefore, unprecedented breakthroughs have been achieved in understanding the nature of cancer and developing new treatment strategies. In this section, we systematically review the four application areas of organoid technology in cancer research. These four areas are: fundamental cancer modeling and mechanistic studies, drug discovery and personalized oncology, immuno-oncology research and therapeutic development, and early cancer detection and translational diagnostics.

### Cancer modeling & Mechanistic studies

**Fig. 3:**
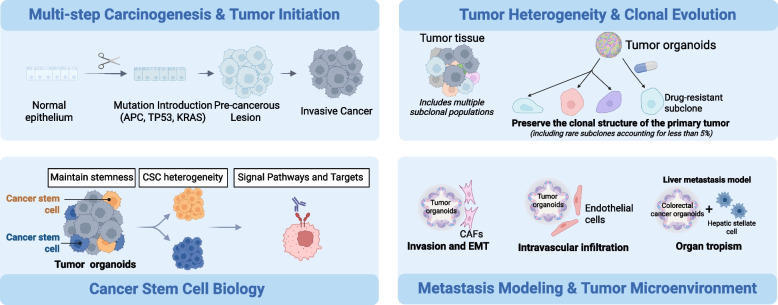
Applications of organoid technology in fundamental cancer modeling and mechanistic studies. Organoids provide a dynamic in vitro platform to dissect the complex biological hallmarks of cancer across four key domains: (1) Multi-step carcinogenesis: Sequential introduction of oncogenic driver mutations (e.g., APC, TP53, KRAS) into normal epithelial organoids enables the step-wise modeling of disease progression from pre-cancerous lesions to invasive cancer. (2) Tumor heterogeneity and clonal evolution: Tumor organoids faithfully preserve the complex subclonal architecture of the primary tumor, including rare subclones (accounting for less than 5%), facilitating the study of clonal dynamics and acquired drug resistance. (3) Cancer stem cell biology: Organoids provide a robust system to investigate CSC stemness maintenance, functional heterogeneity, and targetable signaling pathways. (4) Metastasis modeling and tumor microenvironment: Co-culture systems incorporating CAFs, endothelial cells, and organ-specific stromal cells (e.g., hepatic stellate cells) allow for the functional dissection of invasion, EMT, intravascular infiltration, and organ-specific metastatic tropism. APC: Adenomatous polyposis coli; TP53: Tumor protein p53; KRAS: Kirsten rat sarcoma viral oncogene homolog; CSC: Cancer stem cell; TME: Tumor microenvironment; CAF: Cancer-associated fibroblast; EMT: Epithelial-mesenchymal transition

Organoids have revolutionized our ability to dissect the molecular and cellular basis of cancer initiation, clonal evolution, progression, and metastatic colonization, addressing a critical gap in our understanding of human cancer biology. For decades, our understanding of cancer biology has been largely based on correlative genomic analysis of static human tumor samples, or studies in genetically engineered mouse models (GEMMs), which are limited by interspecies differences between mice and humans. Organoid technology, for the first time, enables researchers to observe and manipulate the dynamic evolution of human tumors in a controlled, physiologically relevant in vitro system, shifting cancer mechanism studies from correlational observations to causal, functional experimentation (Fig. 3). While these foundational advancements have shifted the field toward functional experimentation, the true power of organoid modeling begins with its ability to reconstruct the very first steps of malignant transformation.

#### Modeling multi-step carcinogenesis and tumor initiation

One of the most impactful applications of organoid technology in basic cancer research is the modeling of early tumor initiation and multi-step carcinogenesis. As discussed in Sect. " Gene-Engineered Tumor Organoid Models", gene-edited normal organoid models enable the stepwise introduction of cancer driver mutations, allowing researchers to define the functional consequences of individual mutations, the order of mutation acquisition, and the cooperative interactions between driver mutations during tumorigenesis. These studies have provided direct experimental validation of the multi-step model of carcinogenesis, which was first proposed for colorectal cancer in 1990, but had never been directly tested in a human system prior to the development of organoid technology [101, 115].

Beyond validating existing models of carcinogenesis, organoid studies have revealed novel insights into the earliest stages of tumor initiation, which are nearly impossible to study in human patients, as pre-malignant lesions are rarely resected before progression to invasive cancer. For example, gene-edited gastric organoid models have defined the early driver events in gastric carcinogenesis, showing that CDH1 loss cooperates with TP53 mutation to drive the transition from gastric intestinal metaplasia to invasive gastric adenocarcinoma [116]. Similarly, pancreatic organoid models have revealed that oncogenic KRAS mutation alone is insufficient to drive pancreatic tumorigenesis, and requires concomitant loss of the tumor suppressor CDKN2A to initiate malignant transformation of pancreatic ductal cells [117]. Organoid models have also been used to study the role of germline cancer predisposition mutations in tumor initiation, with iPSC-derived organoids from patients with Li-Fraumeni syndrome (germline TP53 mutation), Lynch syndrome (germline mismatch repair gene mutations), and familial adenomatous polyposis (FAP, germline APC mutation) being used to define the early events of tumor initiation in these high-risk patient populations [118]. However, tumor initiation is merely the starting point; once established, the subsequent diversification of these malignant cells creates a complex landscape of heterogeneity that challenges therapeutic success.

#### Dissecting tumor heterogeneity and clonal evolution

Tumor heterogeneity and clonal evolution are the primary drivers of therapeutic resistance and treatment failure in cancer, yet they remain poorly understood due to the limitations of traditional preclinical models [119]. Organoid technology has emerged as the gold standard platform for studying intratumor heterogeneity and clonal evolution in human cancer, as it uniquely preserves the clonal architecture of the original patient tumor, including rare subclones that are lost in 2D cell line culture.

Single-cell sequencing studies have demonstrated that patient-derived tumor organoids faithfully preserve the complex subclonal architecture and intratumor heterogeneity present in the original primary tumor. This has enabled researchers to perform lineage tracing of clonal evolution under therapeutic pressure, by treating organoids with chemotherapy or targeted therapy and tracking the expansion of pre-existing resistant subclones over time [120]. For example, single-cell multi-omics analysis of colorectal cancer organoids treated with chemotherapy revealed that rare pre-existing subclones with mutations in the DNA damage response pathway drive the emergence of therapeutic resistance, a finding that was subsequently validated in patient tumor samples [121]. Similarly, patient-derived organoid models of head and neck squamous cell carcinoma (HNSCC) have been successfully deployed to track the clonal evolution of resistance to cisplatin. Single-cell RNA sequencing of these models has uncovered a highly specific hybrid EMT program that emerges under treatment, accurately mirroring the transcriptional intratumor heterogeneity observed in clinical patients and identifying novel actionable targets for overcoming resistance [122].

Organoid technology also enables the isolation and expansion of single-cell-derived clonal organoid lines from the same patient tumor, allowing researchers to characterize the genomic, transcriptomic, and phenotypic differences between individual subclones, and define their role in therapeutic resistance, metastasis, and tumor recurrence [123]. These studies have revealed that even minor subclones can have distinct therapeutic vulnerabilities and metastatic potential, highlighting the importance of targeting clonal heterogeneity to prevent treatment failure. Beyond internal clonal shifts, the survival and evolution of these diverse subclones are heavily dictated by their dynamic interplay with the surrounding host environment and their eventual spread to distant sites.

#### Modeling metastasis and tumor microenvironment interactions

Metastasis is responsible for more than 90% of cancer-related deaths, yet it remains the least understood step of tumor progression, due to the lack of in vitro models that faithfully recapitulate the multi-step metastatic cascade [124]. The metastatic cascade is a complex, multi-step process that includes EMT, local invasion, intravasation into the bloodstream or lymphatics, survival in the circulation, extravasation into distant organs, and metastatic colonization, each of which is regulated by complex interactions between tumor cells and the host microenvironment [125, 126]. Traditional 2D culture models are unable to recapitulate these complex, multi-step processes, while GEMMs are limited by interspecies differences and low throughput.

Organoid technology has transformed our ability to model the metastatic cascade in vitro, through the development of multi-cellular co-culture systems that recreate the interactions between tumor cells and the various components of the tumor microenvironment (TME), including cancer-associated fibroblasts (CAFs), endothelial cells, immune cells, and the ECM [127]. Organoid co-cultures with CAFs have been used to study the role of stromal-tumor crosstalk in driving EMT and local invasion, revealing that CAF-secreted cytokines and ECM remodeling enzymes promote the invasive phenotype of tumor cells [128]. Similarly, organoid co-cultures with endothelial cells have been used to model the process of intravasation and extravasation, while organoid-on-a-chip microfluidic systems introduce fluid flow, mechanical tension, and oxygen gradients to mimic the physiological conditions of the vasculature and distant organ microenvironments [129, 130].

Organoid models have also been used to dissect the mechanisms of metastatic organotropism, the process by which tumor cells preferentially metastasize to specific distant organs. For example, co-cultures of colorectal cancer organoids with liver sinusoidal endothelial cells and hepatic stellate cells have been used to model colorectal cancer liver metastasis, the most common site of metastatic spread in colorectal cancer, revealing the critical role of hepatocyte-secreted growth factors in promoting metastatic colonization [131]. Similarly, brain metastasis models have been developed using organoid co-cultures with brain endothelial cells and astrocytes, identifying key signaling pathways that drive the colonization of tumor cells in the brain microenvironment [132]. Crucially, both the primary tumor's growth and its metastatic potential are often driven by a specialized subset of cells that possess unique self-renewal capacities and niche-dependency.

#### Cancer stem cell biology

Cancer stem cells (CSCs), a rare subpopulation of tumor cells with self-renewal capacity and the ability to initiate tumor formation, are thought to be the primary drivers of therapeutic resistance, tumor recurrence, and metastasis [133]. However, CSC biology has been difficult to study in traditional culture systems, which do not support the maintenance of the CSC population in its native niche context. Organoid technology, which replicates the physiological stem cell niche, has emerged as a powerful platform for studying CSC biology in human cancer.

The niche-like culture conditions of organoid systems support the long-term maintenance of CSC populations, preserving their self-renewal capacity and differentiation potential. For example, glioblastoma organoids maintain the cellular hierarchy of the original tumor, including a rare population of glioma stem cells (GSCs) that drive tumor growth and therapeutic resistance [134]. Organoid models have also been used to identify the key signaling pathways that maintain CSC stemness in colorectal, breast, and pancreatic cancer, revealing actionable therapeutic dependencies in the CSC compartment that can be targeted to prevent tumor recurrence [135–137].

Notably, organoid technology has also enabled the functional characterization of CSC heterogeneity, revealing that distinct CSC subpopulations within the same tumor have different metastatic potential and therapeutic vulnerabilities. For example, single-cell-derived clonal organoid lines from breast cancer tumors have identified two distinct CSC subpopulations: one with high metastatic potential and resistance to chemotherapy, and another with high proliferative capacity and sensitivity to targeted therapy, highlighting the need for combination therapies that target both CSC subpopulations to prevent recurrence [112]. Understanding these fundamental biological drivers provides the necessary foundation to translate organoid models from basic research tools into powerful platforms for drug discovery and clinical intervention.

### Drug discovery & Personalized oncology

**Fig. 4:**
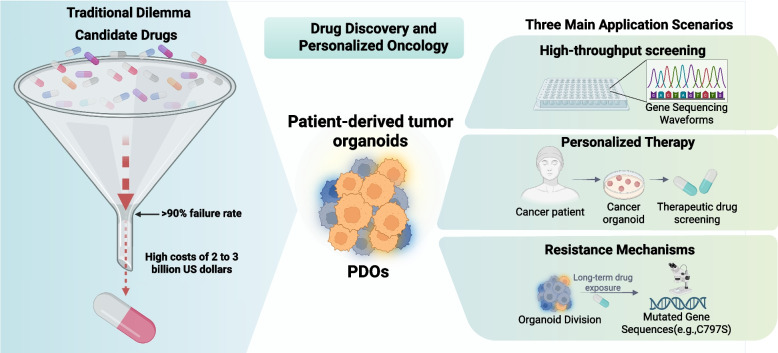
Application of patient-derived tumor organoids (PDOs) in drug discovery and personalized oncology. Conventional cancer drug development suffers from a staggering attrition rate (exceeding 90% failure in clinical trials) and exorbitant costs (estimated at $2–3 billion per approved drug) due to inadequate preclinical models. PDOs effectively bridge this translational gap by providing robust models for three main application scenarios. (1) High-throughput screening: PDO biobanks enable large-scale drug screening coupled with genetic sequencing to identify novel therapeutic targets and predictive biomarkers. (2) Personalized therapy: Tumor organoids established directly from individual cancer patients permit rapid ex vivo therapeutic screening, accurately guiding personalized clinical treatment decisions. (3) Resistance mechanisms: Culturing PDOs under long-term drug exposure faithfully models acquired clinical resistance, facilitating the identification of emergent mutated gene sequences (e.g., the EGFR C797S mutation) and accelerating the development of next-generation therapies. PDO: Patient-derived organoid; EGFR: Epidermal growth factor receptor

The pharmaceutical industry is facing a crisis in cancer drug development, with over 90% of agents that show efficacy in preclinical models failing in human clinical trials, due to the lack of preclinical models that faithfully predict human clinical response. This staggering attrition rate has led to skyrocketing drug development costs, with an estimated $2–$3 billion required to bring a new cancer drug to market, and a slowing rate of new drug approvals despite massive investment. Tumor organoid technology has emerged as a transformative platform for cancer drug discovery and development, bridging the long-standing translational gap between preclinical research and clinical success, while also enabling the clinical implementation of functional precision oncology for individual cancer patients (Fig. 4). To bridge this translational gap at scale, researchers have turned to high-throughput platforms that can represent the vast diversity of the patient population.

#### Organoid biobanks for high-throughput drug screening

The establishment of large-scale living biobanks of patient-derived tumor organoids, spanning hundreds to thousands of patients across dozens of cancer types and molecular subtypes, has transformed the preclinical drug discovery pipeline [138]. Unlike traditional cancer cell line panels, which represent only a small subset of the genomic diversity of human cancer, organoid biobanks capture the full spectrum of genomic, transcriptomic, and histopathological heterogeneity of human cancer, including rare cancer subtypes and molecularly defined patient populations that are underrepresented in cell line panels.

These organoid biobanks enable high-throughput phenotypic screening of approved drugs, investigational agents, and combination regimens in hundreds of genomically annotated patient tumor models, enabling the identification of new predictive biomarkers, genotype-specific therapeutic vulnerabilities, and novel drug targets [139]. For example, a landmark study using a colorectal cancer organoid biobank of over 100 patient lines identified WRN helicase inhibition as a synthetic lethal vulnerability in MSI colorectal cancers, a finding that has since been validated in multiple preclinical models and is now being advanced to phase I/II clinical trials [105, 140]. Similarly, organoid biobanks of rare cancers, such as pediatric brain tumors, rare gynecologic malignancies, and sarcomas, have enabled drug repurposing screens that identified new therapeutic options for these diseases, which have historically been neglected by the pharmaceutical industry due to their low prevalence [141].

Organoid biobanks also play a critical role in the preclinical development of new cancer drugs, enabling the stratification of patient populations that are most likely to respond to a new agent, based on predictive biomarkers identified in organoid screens [142]. This allows pharmaceutical companies to design more efficient clinical trials, enrolling only patients with the relevant biomarker, which significantly increases the likelihood of clinical trial success and reduces the cost of drug development. While large-scale biobanks identify broad therapeutic trends across populations, the ultimate goal of precision oncology remains the selection of the most effective treatment for each individual patient.

#### Patient-derived organoids for functional precision oncology

While genomic profiling has become the standard of care for many advanced cancers, only a minority of patients derive clinical benefit from genomically guided therapy, due to the limited number of actionable genomic alterations, the lack of validated predictive biomarkers for most drugs, and the profound intratumor heterogeneity that drives therapeutic resistance [143, 144]. Patient-derived organoids address this critical limitation by providing a functional readout of a patient's tumor response to therapy, acting as a "living avatar" that can be used to test hundreds of therapeutic regimens ex vivo, to identify the most effective treatment for the individual patient.

Multiple retrospective studies and meta-analyses have confirmed that ex vivo drug sensitivity testing in patient-derived organoids strongly correlates with clinical response in patients across a wide range of cancer types, including colorectal, gastric, breast, pancreatic, lung, and ovarian cancer [145–147]. These studies have consistently shown that organoid testing has a high negative predictive value, meaning that drugs that are inactive in the organoid model are almost universally ineffective in the patient, enabling the elimination of ineffective, toxic therapies that would otherwise be administered to the patient. Organoid testing also has a high positive predictive value, meaning that drugs that are active in the organoid model are likely to induce a clinical response in the patient. Despite the high predictive accuracy of initial drug testing, many patients eventually experience relapse, necessitating a deeper mechanistic understanding of how tumors adapt and survive under therapeutic pressure.

#### Mechanistic studies of therapeutic resistance

Acquired therapeutic resistance is the primary cause of treatment failure and death in patients with advanced cancer, yet the mechanisms of resistance remain poorly understood for most therapies [148]. Tumor organoid models have emerged as a powerful platform for studying the mechanisms of acquired resistance, enabling the generation of isogenic resistant organoid lines via chronic drug exposure, which can be compared to parental sensitive lines using multi-omics profiling to uncover the molecular mechanisms of resistance.

For example, organoid models of EGFR-mutant lung cancer have been used to study acquired resistance to third-generation EGFR inhibitors such as osimertinib, identifying major resistance mechanisms seen in patients, including the EGFR C797S mutation, ECM remodeling, and EMT-mediated resistance [149]. Similarly, organoid models of breast cancer have been used to study acquired resistance to human epidermal growth factor receptor 2 (HER2)-targeted therapy, revealing that increased mechanosignaling and Yes-associated protein 1 (YAP) activation drive resistance to taxane chemotherapy [150]. Organoid models have also been used to study resistance to chemotherapy, immunotherapy, and radiotherapy, identifying novel resistance mechanisms and therapeutic strategies to re-sensitize resistant tumors to therapy [151].

Notably, organoid models enable the study of resistance mechanisms in a patient-specific context, revealing that different patients can develop distinct resistance mechanisms to the same therapy, even if they have the same oncogenic driver mutation [150]. This patient-specific resistance profiling enables the development of personalized combination therapies to overcome acquired resistance in individual patients, a core goal of precision oncology. Importantly, therapeutic resistance is not solely an intrinsic property of tumor cells, but is increasingly recognized as a collaborative process involving the patient’s own immune system.

### Immuno-oncology research

**Fig. 5:**
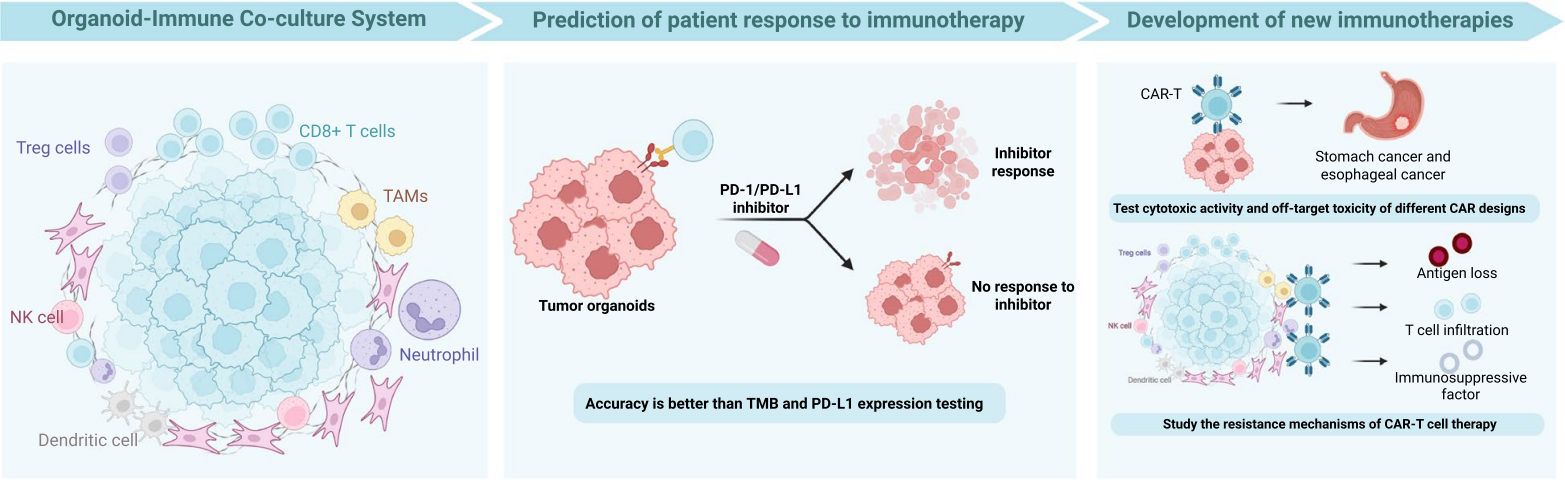
Applications of organoid-immune co-culture systems in immuno-oncology. (1) Organoid-immune co-culture systems: Tumor organoids can be co-cultured with a diverse array of autologous immune cells—including CD8 + T cells, Treg cells, NK cells, TAMs, dendritic cells, and neutrophils—to faithfully reconstruct the complex tumor immune microenvironment (TIME) in vitro. (2) Prediction of clinical response: These co-culture models serve as powerful functional diagnostic tools to evaluate patient-specific responses to immune checkpoint inhibitors (e.g., PD-1/PD-L1 inhibitors). Notably, their predictive accuracy functionally surpasses that of conventional static biomarkers, such as TMB and PD-L1 expression testing. (3) Development of novel immunotherapies: Organoid platforms accelerate the preclinical evaluation of advanced cellular therapies like CAR-T cells (e.g., in gastric and esophageal cancers). They enable rigorous assessment of CAR-T cytotoxic activity, off-target toxicity, and underlying resistance mechanisms, including target antigen loss, insufficient T cell infiltration, and the effects of immunosuppressive factors. Treg: Regulatory T cell; NK: Natural killer; TAM: Tumor-associated macrophage; PD-1: Programmed cell death protein 1; PD-L1: Programmed cell death ligand 1; TMB: Tumor mutational burden; CAR-T: Chimeric antigen receptor T cell

Immunotherapy, particularly immune checkpoint inhibitors (ICIs) targeting the programmed cell death protein 1/programmed cell death ligand 1 (PD-1/PD-L1) and cytotoxic T-lymphocyte-associated protein 4 (CTLA-4) pathways, has transformed the treatment of cancer over the past 15 years, achieving durable long-term responses in a subset of patients with advanced cancer [152]. However, the majority of patients with solid tumors do not respond to ICI therapy, and even in responsive tumor types, most patients eventually develop acquired immune resistance. Progress in immuno-oncology has been severely hindered by the lack of preclinical models that faithfully recapitulate the human tumor immune microenvironment (TIME), autologous tumor-immune crosstalk, and tumor antigen presentation. Traditional preclinical models, including 2D cell lines and immunocompromised mouse PDX models, are completely incompatible with autologous immune modeling, while syngeneic mouse models are limited by interspecies differences in the immune system and tumor biology. Organoid-immune co-culture systems have emerged as a transformative platform for immuno-oncology research, addressing these critical limitations and enabling the development of novel immunotherapies and predictive biomarkers for patient stratification [153] (Fig. 5).The development of these immune-competent models begins with the sophisticated engineering of co-culture systems that can sustain multiple cell lineages simultaneously.

#### Organoid-immune co-culture systems

The core challenge in modeling the human TIME in vitro is the establishment of long-term, functional co-cultures of tumor organoids with autologous immune cells from the same patient, including T cells, tumor-infiltrating lymphocytes (TILs), tumor-associated macrophages (TAMs), dendritic cells (DCs), and other immune cell subsets [154, 155]. Over the past 5 years, multiple robust organoid-immune co-culture platforms have been developed, enabling the study of autologous tumor-immune interactions in a controlled in vitro system.

The first landmark studies in this field demonstrated that co-culture of patient-derived tumor organoids with autologous peripheral blood lymphocytes (PBLs) or TILs could generate tumor-reactive T cells, which specifically killed the tumor organoids while sparing normal tissue organoids from the same patient [156, 157]. These studies showed that the organoid-immune co-culture system preserves the tumor-specific antigen presentation, T cell receptor (TCR) recognition, and cytotoxic function of autologous T cells, mirroring the tumor-immune interactions seen in the patient in vivo. Subsequent studies have expanded these co-culture systems to include other immune cell subsets, including TAMs, CAFs, DCs, and natural killer (NK) cells, creating multi-lineage organoid models that more fully recapitulate the complexity of the TIME [158, 159].

These multi-cellular co-culture systems enable real-time observation of key immune processes, including T cell infiltration into the tumor organoid, T cell activation and expansion, T cell exhaustion, and tumor cell killing via cytotoxic T cells. They also enable the study of the immunosuppressive mechanisms of the TIME, including the role of TAMs and CAFs in creating physical and biochemical barriers that prevent T cell infiltration into the tumor, and the expression of immune checkpoint ligands that drive T cell exhaustion [160]. By successfully capturing these complex interactions in vitro, these co-culture platforms now offer a functional window to predict how a patient’s immune system will respond to modern biological therapies.

#### Predicting immunotherapy response in patients

One of the most impactful clinical applications of organoid-immune co-culture systems is the prediction of clinical response to ICI therapy in individual patients [161]. Current clinical biomarkers for ICI response, including PD-L1 expression, tumor mutational burden (TMB), and MSI status, have limited predictive accuracy, with many patients with high TMB/PD-L1 expression failing to respond to ICI therapy, and many patients with low TMB/PD-L1 expression achieving durable responses [162, 163]. Organoid-based immune reactivity assays address this limitation by providing a functional readout of the patient's autologous T cell response to their tumor, enabling the accurate prediction of ICI response.

Multiple recent studies have demonstrated that organoid-based immune reactivity assays predict clinical response to anti-PD-1/PD-L1 therapy with an accuracy of more than 85%, exceeding the predictive performance of conventional genomic biomarkers. For example, recent advancements have successfully established patient-derived organoid platforms for highly aggressive and immunotherapy-resistant subtypes, such as human mucosal melanoma [164]. Similarly, studies in non-small cell lung cancer, gastric cancer, and hepatocellular carcinoma have shown that organoid assays can identify exceptional responders to ICI therapy who are missed by conventional biomarkers, particularly in patients with mismatch repair proficient (pMMR) tumors with low TMB/PD-L1 expression, who are typically excluded from ICI therapy [165, 166].

These organoid-based immune assays have the potential to transform the clinical use of ICI therapy, enabling the stratification of patients who are most likely to benefit from treatment, while sparing non-responding patients from the toxicities and costs of ineffective ICI therapy. They also enable the identification of patients who may benefit from combination immunotherapies, even if they do not respond to single-agent ICI. Beyond predicting responses to existing drugs, these high-fidelity models serve as an essential proving ground for the next generation of engineered cellular and molecular immunotherapies.

#### Development of novel immunotherapies

Organoid-immune co-culture systems have also emerged as a powerful platform for the preclinical development of novel immunotherapies, including chimeric antigen receptor (CAR)-T cells, T cell engagers (TCEs), bispecific antibodies, antibody–drug conjugates (ADCs), and NK cell therapies [167]. Unlike traditional preclinical models, organoid systems enable the testing of these cellular and antibody-based immunotherapies in patient-matched tumor and normal tissue organoids, enabling the assessment of both on-target tumor killing activity and off-target toxicity against normal tissues, a critical step in the preclinical development of these therapies [168].

For example, organoid models have been used to optimize CAR-T cell constructs targeting Claudin18.2 (CLDN18.2) in gastric and esophageal cancer, testing the cytotoxic activity and off-target toxicity of different CAR designs against patient-matched gastric tumor organoids and normal gastric organoids [167, 169]. Similarly, organoid models have been used to test the efficacy of bispecific T cell engagers (BiTEs) targeting a range of tumor antigens in ovarian, colorectal, and lung cancer, identifying the most potent constructs for clinical development [168]. Organoid systems have also been used to study the mechanisms of resistance to CAR-T cell therapy in solid tumors, including antigen loss, physical barriers to T cell infiltration, and immunosuppressive factors in the TIME, enabling the development of next-generation CAR-T cells that overcome these resistance mechanisms [170].

Notably, organoid biobanks enable the high-throughput screening of novel immunotherapies across hundreds of patient tumor models, enabling the identification of predictive biomarkers of response, and the stratification of patient populations most likely to benefit from the therapy. This is particularly critical for cellular therapies, which are highly personalized and expensive to manufacture, as it enables the design of more efficient clinical trials with higher success rates. While advancing treatments for established tumors remains a priority, the long-term impact on cancer mortality will also depend on applying these diagnostic capabilities to the earliest possible stages of disease.

### Early detection & Translational diagnostics

**Fig. 6:**
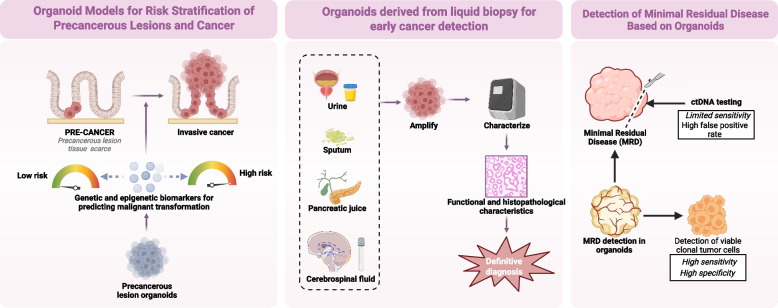
Applications of organoid models in early cancer detection and translational diagnostics. This schematic illustrates three key diagnostic strategies leveraging organoid technology. First, organoid models enable the risk stratification of precancerous lesions (left). By cultivating precancerous lesion organoids from tissue sources, clinicians can analyze genetic and epigenetic biomarkers to predict the likelihood of malignant transformation into invasive cancer, effectively distinguishing between low-risk and high-risk profiles. Second, organoids derived from diverse liquid biopsy sources—such as urine, sputum, pancreatic juice, and cerebrospinal fluid—act as biological amplifiers for early cancer detection (middle). This approach facilitates the in vitro expansion and subsequent functional and histopathological characterization of rare tumor cells to achieve a definitive diagnosis. Finally, the detection of minimal residual disease (MRD) is significantly enhanced using organoids (right). Compared to conventional ctDNA testing, which suffers from limited sensitivity and high false-positive rates, organoid-based MRD detection isolates viable clonal tumor cells, thereby providing superior sensitivity and specificity for clinical evaluation. ctDNA: Circulating tumor DNA; MRD: Minimal residual disease

Early detection of cancer, when the disease is still localized and curable with surgical resection, is the single most effective strategy for reducing cancer-related mortality [171]. However, sensitive and specific early detection tools remain lacking for most cancer types, particularly for "silent" cancers such as pancreatic, ovarian, and lung cancer, which are typically diagnosed at advanced, incurable stages [172, 173]. Current early detection strategies, including imaging and circulating tumor DNA (ctDNA)-based liquid biopsies, have significant limitations: imaging has limited sensitivity for early-stage lesions and high false-positive rates, while ctDNA assays cannot distinguish between viable, clonogenic tumor cells and apoptotic cell debris, leading to false-positive results from clonal hematopoiesis of indeterminate potential (CHIP) and pre-malignant lesions [174, 175]. Organoid technology has emerged as a transformative platform for early cancer detection and translational diagnostics, acting as a biological amplifier that enables the expansion and characterization of rare malignant cells from minimal clinical samples, enabling earlier and more accurate cancer diagnosis (Fig. 6). The effectiveness of these early detection strategies relies first on our ability to model and understand the behavior of lesions before they cross the threshold into invasive malignancy.

#### Organoid models of pre-malignant lesions and cancer risk stratification

Organoid technology enables the establishment of cultures from pre-malignant lesions, including colonic adenomas, gastric intestinal metaplasia, pancreatic intraepithelial neoplasia (PanIN), and bronchial dysplasia, which are rarely available for research due to the lack of clinical indication for surgical resection [176]. These pre-malignant organoid models have enabled the molecular characterization of the stepwise progression from pre-malignancy to invasive cancer, identifying genetic and epigenetic biomarkers that predict malignant transformation, which can be used for cancer risk stratification in high-risk patient populations [177, 178].

For example, recent advancements have demonstrated the feasibility of generating patient-derived organoids directly from endoscopically collected pancreatic juice, providing a novel personalized model for disease modeling [179]. Concurrently, multi-omics profiling of pancreatic juice has successfully identified specific DNA methylation biomarkers that can reliably distinguish pre-malignant PanIN lesions from invasive pancreatic adenocarcinoma, providing a powerful liquid biopsy platform for the identification and surveillance of high-risk patients who may require surgical intervention [180]. Similarly, organoids established from gastric biopsies of patients with intestinal metaplasia have identified biomarkers that predict progression to gastric cancer, enabling risk stratification in patients with chronic Helicobacter pylori infection, the primary risk factor for gastric cancer [181, 182]. These pre-malignant organoid models also enable the testing of chemoprevention strategies, identifying agents that can prevent the progression of pre-malignant lesions to invasive cancer, a critical unmet need in cancer prevention. Transitioning these insights into the clinic requires moving from tissue-based biopsies to less invasive methods that can capture early signals from circulating biofluids.

#### Liquid biopsy-derived organoids for early cancer detection

Liquid biopsy-derived organoids represent a transformative approach for the non-invasive early detection of cancer, enabling the expansion and characterization of rare tumor cells from easily accessible biofluids, including urine, sputum, pancreatic juice, CSF, and even peripheral blood [183]. Unlike ctDNA-based liquid biopsies, which only provide genomic information, organoid cultures derived from biofluids enable the functional and histopathological characterization of the expanded cells, providing a definitive diagnosis of malignancy, while also enabling drug sensitivity testing for patients with screen-detected cancer [184].

Multiple recent studies have demonstrated the clinical utility of this approach across a range of cancer types. For example, patient-derived organoids successfully established from voided urine samples of bladder cancer patients have provided a powerful, non-invasive platform to capture the mutational landscape and intra-tumoral heterogeneity of the original tumor [185]. Rather than serving solely as a diagnostic screen, these urine-derived organoids enable functional drug sensitivity assays, allowing for real-time personalized therapeutic selection that complements standard cytology and genomic profiling [186]. In addition, patient-derived airway organoids established from minimally invasive respiratory samples, such as bronchoalveolar lavage (BAL) fluid or induced sputum, are providing unprecedented in vitro models to study the early stages of lung carcinogenesis in high-risk smokers [87]. While molecular profiling of pancreatic juice collected via endoscopic retrograde cholangiopancreatography (ERCP) or endoscopic ultrasound (EUS) enables high-accuracy early detection, viable cells from these samples can simultaneously be expanded into patient-derived organoids [187, 188]. These organoids provide a vital functional platform for validating early biomarkers and assessing personalized therapeutic vulnerabilities in early-stage pancreatic cancer. For leptomeningeal metastasis (LM)—a devastating complication of advanced cancer that is difficult to diagnose—advanced CSF liquid biopsies and CTC enumerations have achieved diagnostic sensitivities of more than 85%, far outperforming standard cytology. The CSF-derived organoids provide unprecedented in vitro models to explore the unique genomic evolution of LM and conduct personalized drug sensitivity testing for this hard-to-treat metastatic disease [189, 190]. Finally, the diagnostic utility of organoids extends beyond initial detection, providing a critical tool for monitoring the persistence of the disease following curative-intent interventions.

#### Organoid-based minimal residual disease detection

Minimal residual disease (MRD), the presence of rare, viable tumor cells in the body after curative-intent surgery or therapy, is the primary cause of cancer recurrence, which occurs in 30%–50% of patients with early-stage solid tumors after curative-intent treatment [191, 192]. The detection of MRD enables the identification of patients at high risk of recurrence, who may benefit from adjuvant therapy, while sparing low-risk patients from the toxicities of unnecessary adjuvant treatment. Current MRD detection strategies rely primarily on ctDNA assays, which detect tumor-specific mutations in circulating cell-free DNA [193]. While standard ctDNA assays excel at the highly sensitive detection of MRD, they cannot evaluate dynamic tumor vulnerabilities or predict drug efficacy. To overcome this limitation, emerging studies have demonstrated that patient-derived organoids—established from surgical resections or circulating tumor cells—can functionally model residual disease ex vivo. These organoid platforms have shown a strong capacity to predict clinical recurrence and treatment response in patients with gastrointestinal and non-small cell lung cancers, providing a crucial functional complement to ctDNA assays for guiding personalized adjuvant therapy [80, 194].

Organoids have been widely applied in disease modeling, drug screening, immuno-oncology, and early diagnosis. These applications fully demonstrate the significant value of organoid technology in connecting basic research with clinical practice. However, although the clinical transformation prospects of organoids are broad, they still need to overcome some technical challenges before entering routine clinical applications. These challenges mainly include the standardization of operational procedures and the incomplete simulation of the tumor microenvironment.

## Challenges and Perspective

In little more than a decade, organoid technology has evolved from a specialized developmental biology culture method into a cornerstone of modern cancer research and clinical precision oncology. It has addressed long-standing limitations of traditional preclinical cancer models, enabling the faithful recapitulation of human tumor biology, genomic heterogeneity, and therapeutic response in a manipulable, high-throughput in vitro system. Organoid technology has transformed our ability to dissect the fundamental mechanisms of tumor initiation, clonal evolution, metastasis, and therapeutic resistance, while also accelerating the drug development pipeline and enabling the clinical implementation of functional precision oncology for individual cancer patients. From basic mechanistic research to clinical trial design and patient treatment, organoid technology has impacted nearly every area of oncology, and its clinical utility continues to expand at a rapid pace.

Despite these remarkable advances, substantial technical and biological challenges remain that must be addressed to fully realize the clinical potential of organoid technology. The first major challenge is the standardization of organoid culture protocols, media formulations, ECM matrices, and quality control metrics across institutions and laboratories. Currently, there is significant variability in organoid culture protocols between different groups, leading to inconsistent experimental results and limiting the comparability of data between studies [195]. Most protocols still rely on poorly defined, xenogeneic Matrigel as the ECM scaffold, which has significant batch-to-batch variability and is incompatible with clinical-grade applications due to its animal origin [182]. Long-term organoid culture can also lead to clonal selection and genetic drift, potentially reducing the fidelity of the model to the original patient tumor, particularly for late-passage organoid lines [196]. The development of fully defined, xeno-free, synthetic ECM matrices, standardized media formulations, and universal quality control metrics is essential to improve the reproducibility of organoid research and enable the widespread clinical implementation of organoid-based diagnostic assays.

The second major challenge is the incomplete recapitulation of the full complexity of the tumor microenvironment in current organoid models. While significant progress has been made in the development of multi-cellular co-culture systems, few current organoid models support the long-term, functional co-culture of all the components of the native TIME, including functional vasculature, innervation, lymphatics, and dynamic immune cell cycling [197]. Hypoxia, nutrient gradients, mechanical forces, and the biophysical properties of the tumor ECM also remain difficult to replicate in standard static organoid cultures [198]. The integration of organoid technology with bioengineering approaches, including organoid-on-a-chip microfluidic systems, 3D bioprinting, and synthetic biomaterials, will be essential to creating fully physiological, multi-lineage tumor models that accurately recapitulate all aspects of the TIME and enable more accurate prediction of therapeutic response, particularly for immunotherapies [129, 130]. Also, assembloids allow for the controlled integration of diverse tissue types, enabling the study of inter-tissue communication and multicellular interactions that are often lost in simplified 3D cultures [199–201].

The third major challenge is the clinical translation of organoid technology into routine clinical practice. While early prospective clinical trials have demonstrated the clinical utility of organoid-guided therapy, larger, multi-center, randomized phase III clinical trials with standardized clinical endpoints are required to obtain regulatory approval and broad insurance reimbursement for organoid-based diagnostic assays [202]. The clinical implementation of organoid technology also requires the optimization of culture protocols to reduce the time-to-result, which is currently 2–4 weeks for most tumor types, to accommodate patients with rapidly progressive disease who require immediate treatment initiation [203]. The cost of organoid-based testing must also be reduced to make it accessible to all patients, rather than being limited to specialized academic medical centers. The development of automated, high-throughput organoid culture and drug screening systems, enabled by liquid handling robotics and AI-powered image analysis, will be critical to reducing the cost and time-to-result of organoid testing, enabling its widespread clinical implementation [29, 204].

Looking ahead, the integration of organoid technology with cutting-edge genomic, bioengineering, and computational technologies will unlock even greater potential for cancer research and clinical care [205]. The integration of single-cell multi-omics and spatial transcriptomics with organoid models will enable the mapping of clonal architecture, cell-state dynamics, and cell–cell interactions within the tumor ecosystem at unprecedented resolution, revealing novel mechanisms of tumor progression and therapeutic resistance [206]. When combined with genome-wide CRISPR-based functional screening, this will enable the systematic identification of novel cancer driver genes, therapeutic targets, and synthetic lethal interactions, accelerating the development of new cancer therapies [207, 208].

Machine learning and AI will also play an increasingly important role in the future of organoid technology. Predictive models trained on large datasets of organoid drug response and multi-omics profiling data will be able to identify novel genotype-response relationships, optimize combination therapy regimens for individual patients, and stratify patient risk of recurrence and therapeutic resistance [209]. AI-powered automated image analysis of organoid cultures will also enable high-throughput, unbiased assessment of drug response and tumor-immune interactions, eliminating human bias and increasing the scalability of organoid screening [210]. A significant challenge in longitudinal or multi-center organoid studies is the inherent batch-to-batch variability. To ensure that the observed cellular diversity reflects true biological heterogeneity rather than technical noise from 3D culture conditions, robust computational integration is required. Frameworks such as Seurat, Harmony, and LIGER have become indispensable tools for mitigating these batch effects [211–213]. By leveraging these algorithms, researchers can integrate single-cell datasets from various PDO batches, ensuring that the patient-specific digital twin is built upon high-quality, harmonized data that accurately represents the primary tumor’s landscape.

In the coming years, organoid technology will continue to redefine cancer research and clinical care. It will shift cancer drug discovery from murine-centric models to fully human preclinical systems, reducing the staggering attrition rate in clinical drug development and accelerating the approval of new, more effective cancer therapies. It will enable truly personalized cancer care, where every patient's treatment is selected based on functional testing in their own tumor avatar, rather than population-based averages. It will also enable the early detection and interception of cancer, before it progresses to incurable metastatic disease, which will have the greatest impact on reducing cancer-related mortality. Perhaps most importantly, organoid technology reminds us that cancer is not a uniform disease, but a dynamic, patient-specific, evolving ecosystem. By faithfully modeling that ecosystem in the laboratory, we move closer to a future where every cancer patient receives precisely targeted, effective, and minimally toxic therapy, tailored to the unique biology of their tumor.

## Data Availability

Not applicable.
